# Carbon monoxide: impact on remethylation/transsulfuration metabolism and its pathophysiologic implications

**DOI:** 10.1007/s00109-012-0875-2

**Published:** 2012-02-14

**Authors:** Takako Hishiki, Takehiro Yamamoto, Takayuki Morikawa, Akiko Kubo, Mayumi Kajimura, Makoto Suematsu

**Affiliations:** Department of Biochemistry, JST, ERATO, Suematsu Gas Biology Project, School of Medicine, Keio University, 35 Shinanomachi, Shinjuku-ku Tokyo, 160-8582 Japan

**Keywords:** Heme oxygenase, Hydrogen sulfide, Cystathionine β-synthase, Methylation, Glutathione, Cancer, Methionine salvage pathway, Epigenetics, Metabolic systems, Gas biology

## Abstract

Carbon monoxide (CO) is a gaseous product generated by heme oxygenase (HO), which oxidatively degrades heme. While the stress-inducible HO-1 has well been recognized as an anti-oxidative defense mechanism under stress conditions, recent studies suggest that cancer cells utilize the reaction for their survival. HO-2, the constitutive isozyme, also plays protective roles as a tonic regulator for neurovascular function. Although protective roles of the enzyme reaction and CO have extensively been studied, little information is available on the molecular mechanisms by which the gas exerts its biological actions. Recent studies using metabolomics revealed that CO inhibits cystathionine β-synthase (CBS), which generates H_2_S, another gaseous mediator. The CO-dependent CBS inhibition may impact on the remethylation cycle and related metabolic pathways including the methionine salvage pathway and polyamine synthesis. This review focuses on the gas-responsive regulation of metabolic systems, particularly the remethylation and transsulfuration pathways, and their putative implications for cancer and ischemic diseases.

Heme oxygenase (HO) degrades protoheme-IX by cleaving its α-methene bridge into free divalent iron (Fe^2+^), biliverdin-IXα, and carbon monoxide (CO) [1–4]. The reaction uses nicotinamide adenine dinucleotide phosphate (NADPH)-cytochrome P450 reductase as an electron donor system [5, 6] and O_2_ as the acceptor [7]. In humans, nearly 80% of the bilirubin in bile derives from hemoglobin heme. Cytochromes P450 constitute another major source of heme that undergoes the HO-dependent degradation. Products of the HO reaction were regarded as potentially toxic wastes destined only for excretion. However, this changed when evidence emerged for physiologic roles of bilirubin-IXα, a potent anti-oxidant generated through biliverdin reductase [8–11].

The HO/CO system serves as a neurovascular regulator, as CO has a modest ability to activate soluble guanylate cyclase [12], a receptor of NO [13, 14]. However, a variety of biological responses specifically triggered by CO have attracted attention and led to a series of macromolecular receptors for CO that do not respond to NO. This article focuses on the interaction between the HO/CO system and cystathionine β-synthase (CBS), which is a rate-limiting enzyme regulating methionine metabolism and transsulfuration pathway that serves as an H_2_S-generating system [15–19].

## Identification of cystathionine β-synthase as a CO-regulated protein by metabolomics

The transcriptional activator CooA in the photosynthetic bacteria *Rhodospirillum rubrum* is the first example of a heme protein in which CO plays a physiological role [20]. Only the CO-bound form of CooA binds to its target DNA and acts as a transcriptional activator [20–22]. In mammals, the heme protein neuronal PAS domain protein 2 (NPAS2) was reported to be a specific CO sensor [23, 24]. NPAS2 was identified as a member of the basic-helix-loop-helix (bHLH) family of transcription factors expressed in the forebrain. The resonance Raman spectra indicated that CO coordinated to the heme iron histidine on the proximal side, whereas NO did not bind to the heme group [25]. CO has been proposed to regulate the formation of a complex between NPAS2 and BMAL1, another bHLH transcription factor, that regulates the circadian rhythms [23].

Many heme enzymes including cytochromes P450 were once considered putative CO-sensitive signal transducers [26–28]. However, the ferrous heme of these enzymes has been found to be sensitive to both CO and NO, ruling them out as CO-specific sensors. On the other hand, CBS, the “pseudo-cytochrome P450”, was found to be a strong candidate for a CO-specific sensor. In vitro studies using recombinant CBS reported that CO, but not NO, acted as a competitive inhibitor of CBS [27, 29] with the *K*_i_ value of several micromolar, much smaller than that for NO (200 μM). CO inhibits recombinant rat CBS by stabilizing the 6-coordinated structure of the heme. By comparison, NO binds to heme, but stabilizes the 5-coordinated structure. CBS was first identified as H450 where the addition of CO to its reduced form produced a new spectral species that resembled that of the reduced CO complex of a denatured form of cytochrome P450 [30]. Among heme proteins, CBS is unique in that it catalyzes a pyridoxal phosphate (PLP)-dependent reaction [31]. The prosthetic heme of this enzyme is coordinated to histidine and cysteine as axial ligands in human and rodents. Although the crystallographic structure of CO-ligated forms has yet to be determined, perturbation of the heme environment by CO, but not by NO, is believed to be communicated to the active site with concomitant inhibition of enzyme activity. Low *K*_i_ for CO suggests that CBS acts as a CO-specific sensor under physiological conditions.

**Fig. 1:**
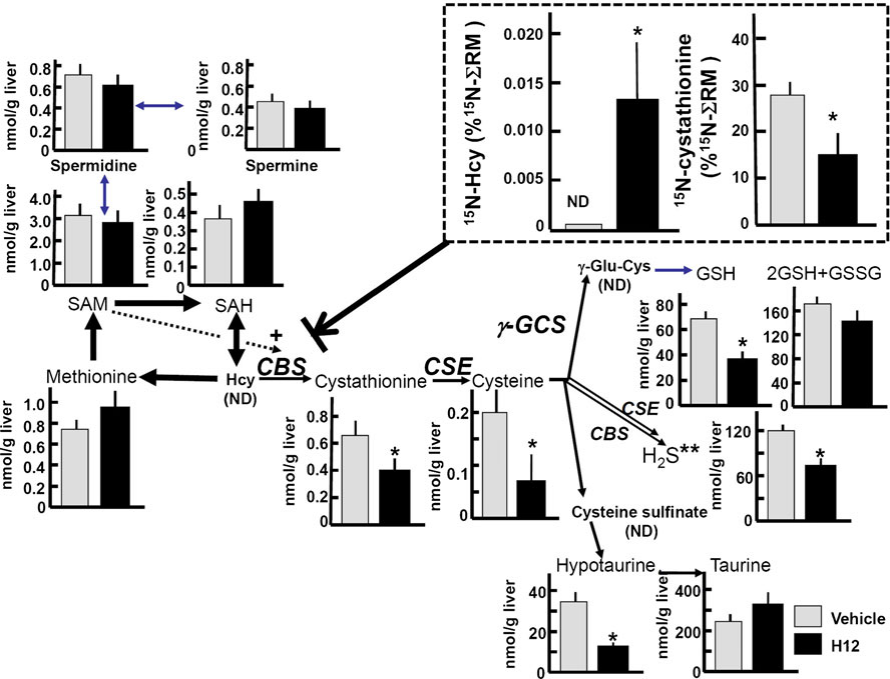
Differential metabolomics reveals that CO upregulates metabolites in remethylation cycle and downregulates those in transsulfuration pathway. Metabolomic comparison of sulfur-containing amino acids and their derivatives between the heme-overloaded and vehicle-treated livers of mice. Differences in hepatic contents of the metabolites between the control and hemin-treated mice. *H12* treatment with hemin at 12 h prior to sampling the liver. Note decreases in transsulfuration metabolites. In vivo pulse-chase analysis indicating conversion rates of ^15^N-methionine into ^15^N-homocysteine (Hcy) and ^15^N-cystathionine in livers between the groups (*dotted square*). The amounts of the downstream metabolites were measured at 30 min after the methionine administration. The data in the *dotted square* were normalized by total amounts of metabolites in remethylation cycle (^15^N-methionine + ^15^N-SAM + ^15^N-SAH + ^15^N-Hcy = ΣRM) at 30 min. *ND* not detected. Data indicate mean ± SE of six to eight separate experiments for each group. *P < 0.05, compared to the vehicle-treated group. Adapted by permission from Wiley: Shintani et al. Hepatology, 49: 141–150, 2009 [29]

**Fig. 2:**
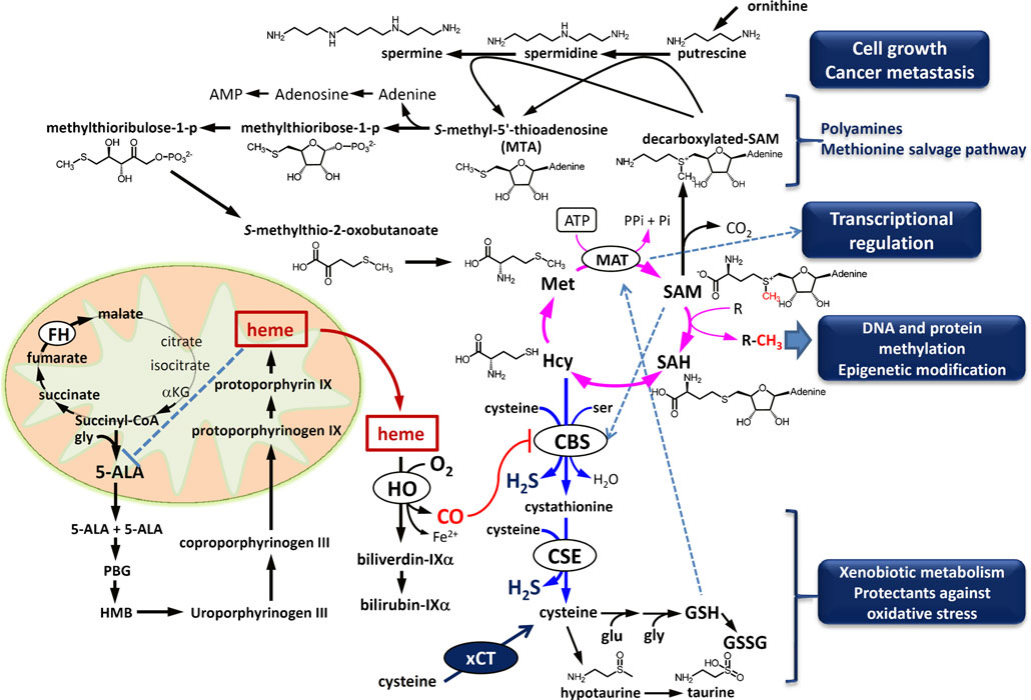
Possible metabolic pathways modulated by a CO-sensitive CBS inhibition. Not only does CBS inhibition by CO alter remethylation cycle (*blue arrows*) and transsulfuration pathway (*pink arrows*) but it may also modulate methionine salvage pathway and polyamine metabolism. *Dotted arrows* and a *line* indicate activation and inhibition of corresponding enzymes by metabolites, respectively. *ALAS* aminolevulinic acid synthase, *FH* fumarate hydratase, *CSE* cystathionine γ-lyase, *MAT* methionine adenosyl transferase, *αKG* α-ketoglutarate, *ALA* aminolevulinic acid, *PBG* porphobilinogen, *Hcy* homocysteine, *SAM S*-adenosylmethionine, *SAH S*-adenosylhomocysteine

Gases have the ability to bind to metal-centered prosthetic groups of many proteins. It is likely that gas messengers alter the activity of enzymes with metal-centered prosthetic groups. To test this possibility, we applied metabolome analyses using capillary electrophoresis assisted by mass spectrometry (CE–MS) to search for a candidate enzyme responding to CO in vivo [29]. In these studies, differential metabolomics display suggested that CO upregulates metabolites in the remethylation cycle and downregulates those in the transsulfuration pathway (Fig. 1). In vivo pulse-chase analysis of ^15^N-methionine in livers of control mice and hemin-treated mice in which HO-1 is induced revealed accumulation of ^15^N-homocysteine and suppression of ^15^N-cystathionine under the CO-overproducing conditions, suggesting that metabolic flux through CBS is suppressed by CO. The ability of CO to limit CBS activity as a regulator of the transsulfuration pathway may have diverse impacts on biological systems. As seen in Fig. 2, the HO/CO system stands between the tricarboxylic acid (TCA) cycle and methionine/thiol metabolism. In the following sections, we will discuss pathophysiological consequences brought about by altered metabolic flux in the transsulfuration pathway by the HO/CO system.

## CO-sensitive CBS and methionine metabolism in cancer

A growing body of evidence suggests that HO-1 may play a role in tumor induction and can potently increase the growth and spread of tumors. HO-1 expression is often increased in tumor tissues and is further elevated in response to radio-, chemo-, or photodynamic therapy [32]. At present, whether inhibition or induction of HO-1 aggravates development of cancer remains controversial, and prognosis in experiments using cancer cell lines highly depends on the particular cell lines that are analyzed. For instance, in recent studies using HepG2 cells [33], overexpression of wild-type HO-1, but not that of mutant HO-1, decreased the migration of the cancer cells. They have shown that HO-1 plays an important role in hepatocellular carcinoma progression through p38MAPK activation both in vivo and in vitro conditions. On the other hand, HO-1/CO system serves as an anti-apoptotic signal followed by activation of Ras–Raf–ERK system that triggers nrf2 to upregulate the enzyme [34]. Recently, Otterbein et al. [35] showed that HO-1/CO pathway participates in the DNA-repair process. Naïve *Hmox-*null mice exhibit excessive tissue levels of γ-histone H2A (γ-H2AX), a marker of ongoing and chronic DNA damage. In addition, administration of genotoxic stressors in HO-1-deficient fibroblasts resulted in no γ-H2AX foci formation or phosphorylation of γ-H2AX. In this model, HO-1 induction or CO treatment induced homologous recombination-mediated DNA repair through the activation of the DNA repair kinases, ataxia telangiectasia-mutated (ATM) and ataxia-telangiectasia rad3-related (ATR), while lack of HO-1 resulted in the inhibition of phosphorylation of ATM and ATR. Direct molecular target(s) that allow CO binding remains unclear in this study.

Through multiple mechanisms, cancer cells forming tumor might generate considerable amounts of CO through HO. Recent studies using renal cell carcinoma cells showed roles of mutant fumarate hydratase (FH, Fig. 2) in their development. FH is an enzyme of the TCA cycle that catalyzes the hydration of fumarate to malate. The germline mutation of FH leads to the accumulation of fumarate and the upstream TCA intermediates in the cells [36]. However, no mechanism has been provided to explain the ability of the cell survival without cycling all the TCA intermediates of this metabolic hub. Frezza et al. [36] used genetically modified kidney mouse cells where Fh1 was deleted, and conducted a metabolic simulation in silico to predict and validate a linear metabolic pathway starting from glutaminolysis and ending with HO-dependent heme degradation and bilirubin formation. This pathway, which involves the biosynthesis and degradation of heme, enables Fh1-deficient cells to use the metabolites of TCA cycle and permits partial mitochondrial NADH production necessary to maintain ATP. They confirmed that targeting this pathway would render Fh1-deficient cells non-viable, while sparing wild-type Fh1-expressing cells. Considering the presence of various mutant TCA enzymes among different types of cancer cells, targeting an aberrant branching pathway might contribute to suppression of the cancer growth and development. At the same time, drawing off TCA intermediates to heme synthesis and degradation serves as a stratagem for survival of certain types of cancer cells.

**Fig. 3:**
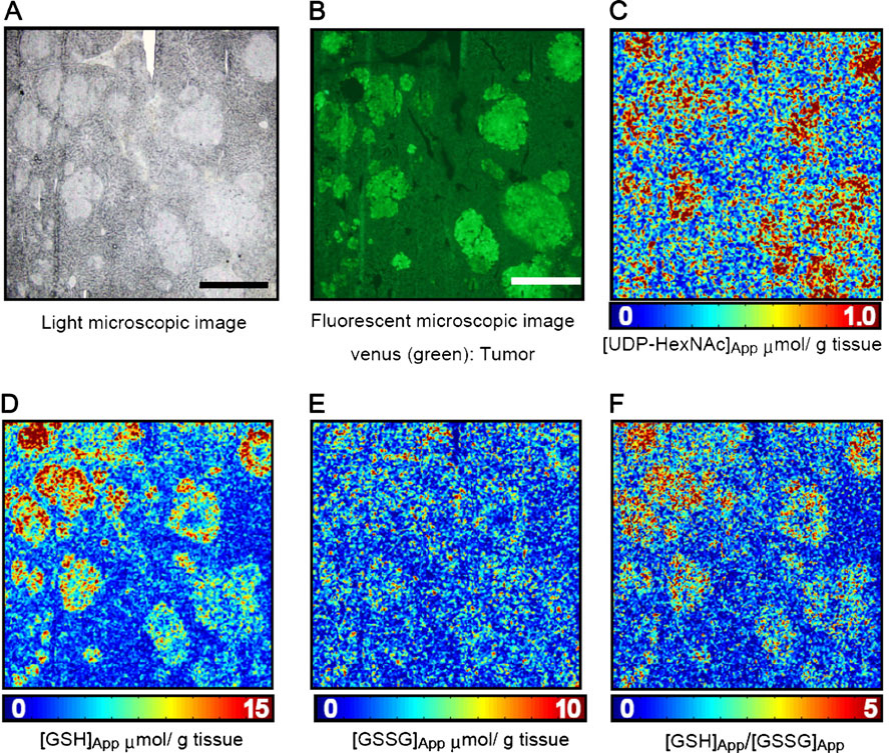
Glutathione and UDP-HexNAc as marker metabolites enriched in colon cancer metastasis. **a** Light-microscopic photograph of intrasplenically injected HCT116 colon cancer cell xenografts in the liver of NOG mice. *Scale bar*: 500 μm. **b** A green fluorescence image of the same specimen shown in (**a**). **c**–**f** Representative imaging mass spectrometry showing spatial distribution of apparent UDP-HexNAc concentration (UDP-HexNAc_app_), the reduced type of glutathione (GSH_app_), oxidized glutathione (GSSG_app_), and (GSH_app_)/(GSSG_app_) ratio in the same microscopic field plotted as a heat map, respectively. Adapted by permission from Springer: Kubo et al. Anal Bioanal Chem, 400: 1895–1904, 2011 [43]

Upregulation of HO might benefit survival and development through multiple pathways. First, activated heme degradation to provide bilirubin serves as a potent anti-oxidant that actually accounts for the protective mechanism for human neuroblastoma cells that became resistant to GSH-depleting reagents [37, 38]. Second, CO-dependent inhibition of CBS in cancer cell lines triggers upregulation of remethylation metabolites [e.g., methionine (Met), *S*-adenosylmethionine (SAM), *S*-adenosylhomocysteine (SAH) in Fig. 2]; changes in these metabolites determine cellular contents of polyamines that regulate cancer metastasis, and regulate protein methylation and epigenetics [39–41]. On the other hand, CBS inhibition by CO acutely downregulates transsulfuration metabolites which is followed by recovery of cysteine and GSH, presumably because of compensatory upregulation of xCT (cystine transporter) that salvages cystine as a precursor to generate these anti-oxidants. Ishimoto et al. have recently reported a role for variant CD44, a receptor for hyaluronic acid, in stabilizing xCT in the cell membrane that helps GSH accumulation for the cell survival [42]. Upregulation of GSH in tumor also occurs under in vivo conditions (Fig. 3) [43].

As mentioned above, CO acutely causes a decrease in the GSH amount through its inhibitory action on CBS, as depicted in Fig. 1. However, CO is known to shift glucose utilization toward pentose phosphate pathway, thereby providing high NADPH to recycle GSSG to its reduced form GSH [44], while molecular mechanism underlying this metabolic shift remains to be solved. Increases in remethylation metabolites by stress-inducible CO might regulate histone methylation to confirm cancer cell survival [39]. In human U937 monoblastic leukemia cells, knocking down CBS diminishes the responsiveness to CO or HO-1 induction. These results suggest that CO-sensitive CBS system controls protein methylation which is implicated in epigenetic regulation.

Recent studies by Katoh et al. [45] revealed a novel role of methionine adenosyl transferase (MAT) located in nuclei. They performed proteomics analysis of MafK, revealing its interaction with MATIIα, a MAT isozyme. MATIIα was localized in nuclei and found to form a dense network with chromatin-related proteins including Swi/Snf and NuRD complexes. MATIIα was recruited to the Maf recognition element (MARE) at the HO-1 gene. When MATIIα was knocked down in a murine hepatoma cell line, expression of HO-1 was repressed at both basal and induced levels. The catalytic activity of MATIIα, as well as its interacting factors such as MATIIβ, BAF53a, CHD4, and PARP1, was required for HO-1 repression. MATII thus serves as a transcriptional corepressor of MafK by interacting with chromatin regulators and supplying SAM for methyltransferases. Nuclear translocation of enzymatically intact MATII causes HO-1 repression, while its mutant expression or loss of MATII causes significant induction of HO-1 through impaired histone methylation at HO-1 locus. Implication of this novel mechanism for regulation of cancer growth and development should deserve further studies.

## CO-dependent H_2_S inhibition: roles in pathophysiology

Recent studies revealed that bacteria normally producing H_2_S acquire antibiotic resistance through mechanisms mitigating oxidative stress imposed by antibiotics [46]. Considering that H_2_S has the ability to neutralize electrophilic compounds, it is not unreasonable to hypothesize that the gas has the ability to detoxify anti-cancer reagents, some of which are converted to electrophiles in the body. In other words, selective delivery of such compounds might ameliorate toxicity of anti-cancer reagents against the host tissues. Recent studies provided evidence that H_2_S-donating reagents have the potential to suppress cancer development [47]. Therefore, it is likely that upregulation of HO/CO system might facilitate tumor growth in part by suppressing CBS-derived H_2_S.

CO-sensitive CBS/H_2_S system plays crucial roles in homeostasis of organ functions. Several lines of evidence support the concept that CBS acts as an in vivo CO sensor. In the mouse liver, the low-end value of endogenous CO is approximately 5 pmol CO/mg tissue [48], suggesting that tissue concentration of CO is in the micromolar range. Murine hepatocytes express both CO-producing HO-2 [49] and H_2_S-producing CBS. In addition, HO-1 is induced in both hepatocytes and Kupffer cells under stress or disease conditions [50]. The close proximity of the enzyme distributions taken together with measured CO concentrations, and the kinetics of CBS activity suggests that the enzyme is acting as a CO sensor [51]. Shintani et al. [29] demonstrated that an increase in hepatic CO content causes global decrease in transsulfuration metabolites such as cystathionine, cysteine, and hypotaurine, as shown in Fig. 1. Administration of a stress-inducible level of CO (as 20 μmol/kg of CO-releasing molecule) caused a decrease in hepatic H_2_S content to stimulate HCO_3_^−^-dependent biliary choleresis. Such a CO-sensitive metabolic adaptation might play a regulatory role in biliary excretion that facilitates the solubility of xenobiotic metabolites under disease conditions or detoxification processes [29, 52–55].

**Fig. 4:**
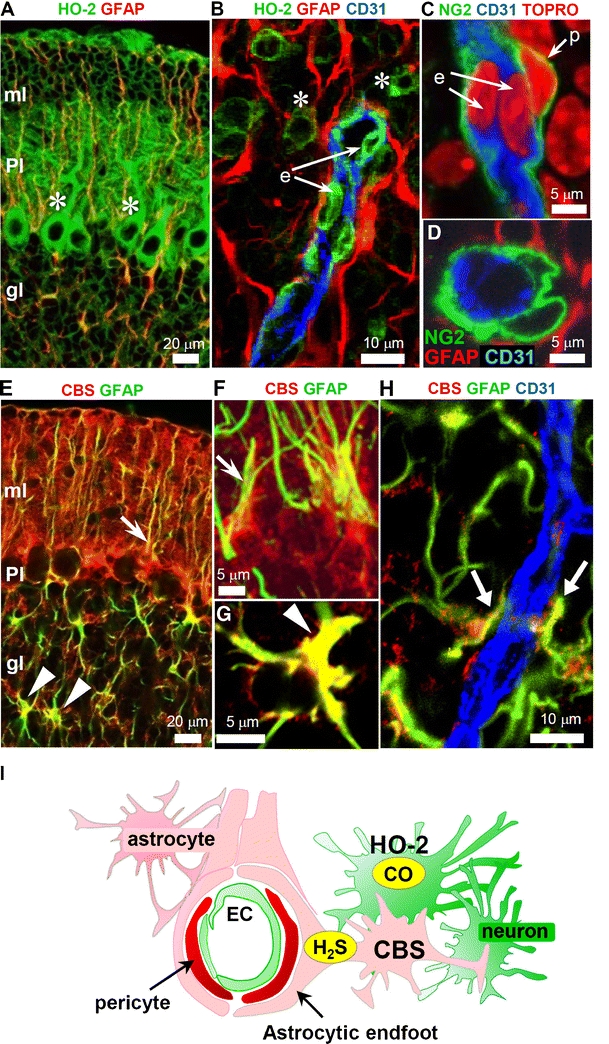
Immunohistochemical localization of HO-2 and CBS in the neurovascular unit of neonatal mouse cerebellar cortex. Neurons and endothelial cells express HO-2 (**a**, **b**), the constitutive CO-producing enzyme, whereas glial cells express CBS (**e**–**h**), an H_2_S producing enzyme. Note that HO-2-positive cells along vessel wall are endothelial in (**b**), not pericytic, since nuclei of NG2 (pericytic marker) positive cells, stained with TO-PRO-3 (a nucleic acid stain), are completely devoid of CD31 (endothelial marker) labeling in (**c**). The arteriolar wall is surrounded by NG2-positive pericytes in (**d**), key contractile cells within the neurovascular unit. **i** Schematic depiction of the localization of HO-2 and CBS in the neurovascular unit. *GFAP* glial fibrillary acidic protein, an established marker of glial cells; *ml* molecular layer; *Pl* Purkinje cell layer; *gl* granular layer; *e* endothelium; *p* pericyte. Adapted by permission from National Academy of Sciences, USA: Morikawa et al. PNAS, 109: 1293–1298, 2012 [68]

Hypoxia alters CO-sensitive CBS/H_2_S pathway to trigger acute adaptive responses for maintaining homeostasis. Although the brain is the most susceptible organ to O_2_ deprivation, it can increase blood flow in response to hypoxia by several mechanisms. They include potassium [56], adenosine [57, 58], hydrogen ions [59], lactate, and prostaglandin E_2_ [60]. These adaptive responses are critical for delivery of O_2_ and cellular transport of glucose in brain tissue. Readers are referred to an excellent review by Attwell et al. [61] for more comprehensive account on this subject. Here we focus on a gas-mediated cascade that plays a key role in regulating blood flow and energy metabolism. Brain generates large amounts of CO mainly from constitutive HO-2 reactions [62]. CO is known to regulate neuronal transmission [9, 63]; however, physiologic roles of CO in the central nervous system have not been fully understood. In the peripheral nervous systems, HO-2 is an O_2_ sensor in the carotid body, an organ responsible for sensing O_2_ levels in arterial blood [64, 65]. Because HO-2 requires molecular O_2_ for its activity, it has been proposed that stimulation of carotid body action by hypoxia may reflect in part reduced formation of CO [66]. A recent study reported that H_2_S mediates carotid body stimulation by hypoxia and hypoxia-evoked H_2_S generation in the carotid body requires interaction of CSE and HO-2, which generates CO [67] [see Prabhaker NR and Semenza GL, this volume]. In the central nervous system, Morikawa et al. [68] recently demonstrated that HO-2 can function as an O_2_ sensor in the brain and an O_2_–CO–H_2_S cascade rapidly mediates hypoxia-induced cerebral vasodilation. In this cascade, hypoxia elicits vasodilation via the coordinate actions of CO generated by HO-2 and H_2_S generated by CBS, similar to that proposed in the carotid body. Pericytes, major contractile cells that control the diameter of microvessels [61, 69], are surrounded by CO- and H_2_S-producing cells (Fig. 4). By actually measuring tissue CO content, these authors showed that HO-2 synthesizes a fair amount of CO under normoxia and hypoxia reduces CO production. Since CO tonically inhibits CBS, hypoxia releases the tonic inhibition leading to increased levels of H_2_S derived from the enzyme that mediate the vasodilation [70] of small arterioles. Such hypoxia-induced vasodilation of arterioles does not occur in CBS-null mice while remaining intact in CSE-null mice [68].

**Fig. 5:**
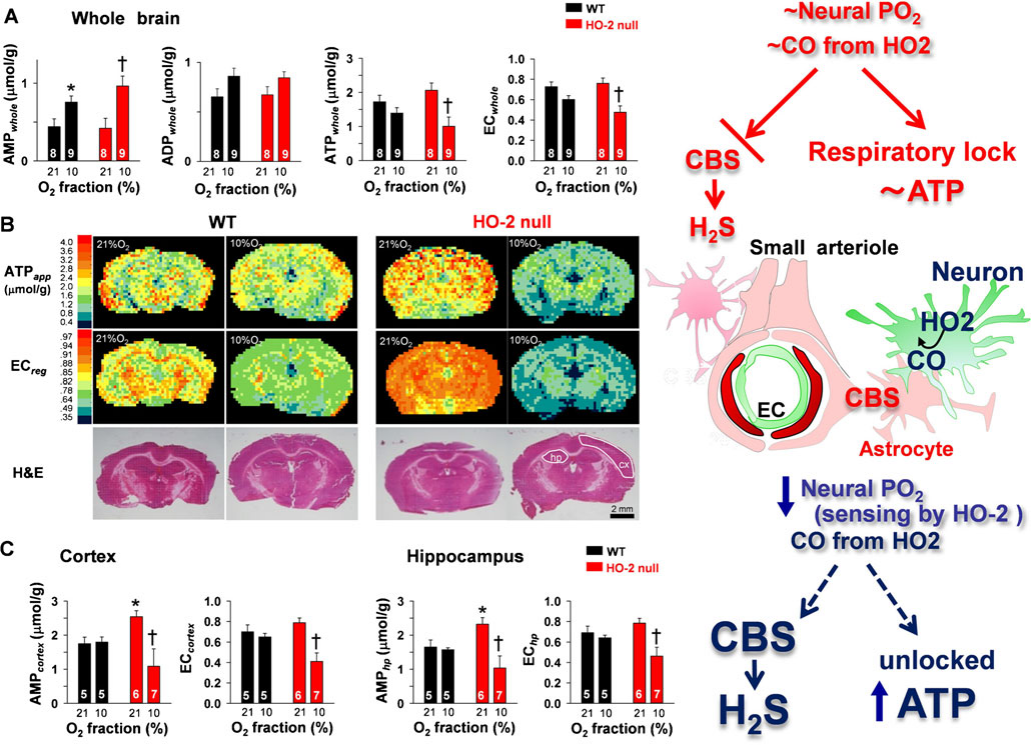
Impaired ability of HO-2-null mice to maintain ATP levels on exposure to 10% O_2_ for 1 min. **a** Alterations in AMP (AMP_whole_), ADP (ADP_whole_), ATP (ATP_whole_), and energy charge (EC_whole_) in the whole brain. *P < 0.05, compared to WT normoxia. †*P* < 0.05, compared to HO-2 null normoxia. **b** Representative imaging mass spectrometry showing spatial distribution of apparent ATP concentration (ATP_app_) and energy charge (EC_reg_). Note the basal increase in ATP in HO-2-null mice. *Bottom panels*—H&E staining after imaging mass spectrometry. *cx* cortex, *hp* hippocampus. **c** Quantitative analysis of regional ATP concentration and energy charge in WT and HO-2-null mice. *P < 0.05, compared to WT normoxia. †*P* < 0.05, compared to HO-2-null normoxia. Adapted by permission from National Academy of Sciences, USA: Morikawa et al. PNAS, 109: 1293–1298, 2012 [68]

Physiologic consequences of HO-2 loss are intriguing. Namely, basal ATP content in the brain is increased by the deletion of HO-2, suggesting that CO mildly suppresses ATP production during normoxia. Once the tonic inhibition is relieved by hypoxia, it gives way to the rise in dynamic strength of compensatory ATP maintenance. In HO-2-null mice, neurovascular units lacking such a tonic inhibitory system are unable to compensate ATP levels upon hypoxia (Fig. 5). Such a notion is consistent with previous studies indicating that pharmacological inhibition of HO increases the basal O_2_ consumption in the liver [71] and that an increase in endogenous CO by the enzyme induction inhibits cellular respiration through its inhibitory effects on cytochrome *c* oxidase [72]. Although the study provides evidence for a novel protective mechanism of neurovascular units against hypoxia that is operated by multiple gases, further investigation is required to reveal functional links between neuronal and microvascular coupling through multiple gases and gas-responsive metabolic systems.

## Conclusion

Stress-inducible and constitutive CO plays physiologic roles for maintaining homeostasis of cell and organ function. CO binding to CBS impacts remethylation and transsulfuration pathways, leading to regulation of protein methylation involving epigenetic modification and downregulation of H_2_S, and resultant alterations in cell functions in vivo, respectively. Identification of methylated molecular targets and/or macromolecules responsible for direct H_2_S binding is needed to establish a concept that gases such as O_2_, CO, and H_2_S constitute an important class of messengers that regulate metabolic systems through multiple mechanisms.
